# Role of Plasticizers on PHB/bio-TPE Blends Compatibilized by Reactive Extrusion

**DOI:** 10.3390/ma15031226

**Published:** 2022-02-07

**Authors:** Kerly Samaniego, Armando Matos, Estefanía Sánchez-Safont, María V. Candal, Jose M. Lagaron, Luis Cabedo, Jose Gamez-Perez

**Affiliations:** 1 Polymers and Advanced Materials Group (PIMA), Universitat Jaume I, 12071 Castelló de la Plana, Spain; samanieg@uji.es (K.S.); amatos@uji.es (A.M.); esafont@uji.es (E.S.-S.); lcabedo@uji.es (L.C.); 2 School of Engineering, Science and Technology, Valencian International University (VIU), 46002 Valencia, Spain; mariavirginiacandal@campusviu.es; 3 Novel Materials and Nanotechnology Group, Institute of Agrochemistry and Food Technology (IATA), Spanish National Research Council (CSIC), 46980 Valencia, Spain; lagaron@iata.csic.es

**Keywords:** PHB, elastomer, compatibilizer, thermoforming

## Abstract

Poly(hydroxybutyrate) (PHB) is a biopolymer biologically synthesized by controlled bacterial fermentation from a wide variety of microorganisms. PHB is proposed as a potential green alternative to commonly used plastics in packaging, due to its biodegradability and biocompatibility. However, if PHB is to replace commodities, it has some limitations regarding its thermo-mechanical performance to overcome. Among them are its critically the low toughness values at room temperature and poor thermoforming ability. With the aim of overcoming these weaknesses, in this work, blends of PHB with the addition of a biodegradable thermoplastic elastomer (bio-TPE) were prepared and evaluated. Films of such compounds were made by cast extrusion. In order to enhance the compatibility of both polymers during the extrusion process, three different reactive agents (poly-hexametylene diisocianate, triglycidyl isocyanurate, and Joncryl^®^ ADR-4368) were assessed. The morphology and mechanical- and thermal properties of the films obtained were analyzed. In addition, the thermoforming ability of the produced films was evaluated. The results show that the plasticizers present in the bio-TPE interacted with the reactive agents, making them chemical competitors and altering the outcome of the blends.

## 1. Introduction

The social pressure on reducing plastic pollution is leading researchers and industry players to attempt to find biodegradable biobased plastics that could replace commodities in single-use and short-life applications [1].

Plastics have become essential materials in modern life, finding applications in almost all industrial fields, such as packaging, building and construction, automotive, consumer electronics, and toys. However, at present, most plastic materials are fossil-based and are produced from oil or gas. Indeed, the polymers used are mostly non-biodegradable, which causes pollution when the residues are not handled properly. As these polymers are cheap, recycling these residues is not always a viable option, and on many occasions they end up in dumping sites or at the sea, where they become pollutants [2]. Hence, there is interest in the development of alternatives that may be more environmentally friendly, especially in applications with short life and/or single use, such as packaging. The packaging market is the largest field of application for plastic bio-materials, with a 47 percent share of destination from the total production of bioplastics in 2020 [3].

Within this context, bio-sourced polyesters have received great attention, especially those that are biodegradable. They offer the opportunity to overcome some of the problems derived from waste management [4,5]. Among them, Poly(hydroxyalcanoates) (PHAs) have gained significant attention by researchers and by industry players, because PHAs are high-molecular-weight polymers biologically synthesized by a wide variety of microorganism such as bacteria, when they are submitted to particular stressful feeding conditions [6]. In that way, PHAs are a potential source of bioplastics that can be produced from organic waste, reducing the carbon footprint, and enhancing circular economy [7,8].

Generally, PHAs can be isotactic semicrystalline high-molecular weight thermoplastic polymers, with physical and mechanical properties similar to some conventional plastics, but from renewable resources. They are biodegradable and biocompatible [9], have low permeability to water (depending on their composition), and are stable against UV rays. PHAs have great potential in applications in the packaging sector [8,10]. They cover a wide range of mechanical and thermal properties, since they can be produced with different repetitive units (butyrate, valerate, hexanoate…) and copolymers with different content of their repeating units [11]. For instance, poly(hydroxybutyrate) (PHB), has a melting point and glass transition temperature similar to those of Polypropylene (PP) and mechanical performance and barrier to similar to gases such as polyethylene terephthalate (PET). Random copolymers of PHB with valerate (PHBV) or Hexanoate are able to modify the thermomechanical behavior of PHB [12,13].

Due to its biodegradability, biocompatibility, and its manufacture from renewable resources, PHB is currently of great interest for rigid trades in food packaging applications, as those products have a single use, short life, and end up contaminated with organic waste [14,15,16]. However, pristine PHB presents some critical weaknesses that limit its industrial applicability. These are low elongation-at-break, low toughness (and tear resistance), a narrow processing temperature window, and high production cost. Some of these drawbacks are related with the high crystallinity ratio that is developed during cooling from the melt, and the fact that the melting temperature of such crystalline phase is close to the degradation temperature of PHB [17,18]. Furthermore, at room temperature, physical aging with secondary crystallization phenomenon takes place in PHB, which changes the mechanical properties and embrittles the material [19,20,21].

It is well known that the addition of an elastomeric phase to rigid polymer can improve the toughness of the latter (rubber toughening process). The elastomeric phase can absorb the deformation via multiple cavitation, this phenomenon being improved by a good dispersion of the elastomeric phase in the rigid matrix and good interfacial interaction between both phases [22,23]. PHB can so be improved by blending it with a thermoplastic elastomer (such as polyurethane), as shown in a previous work [24].

In this case we considered the possibility of obtaining a 100% biosourced plastic, based on PHB, by blending it with a biobased thermoplastic elastomer (bio-TPE). This elastomer had to be selected among those with suitable processing temperatures, capable to match that of PHB. That means that it must be fluid at 180 °C, since processing temperatures above this one would cause thermal degradation of PHB. Furthermore, the viscosity of such bio-TPE at the processing temperatures has to be adequate to produce an efficient shear to get a fine dispersion in the matrix [25].

To better match two or more phases in blends, compatibilizers are commonly used. They help to improve the interaction between the secondary phase and the matrix, producing a decrease in the size of the particles and reducing the cavitation distance [22]. One way to achieve this is to compatibilize both phases by reactive extrusion, that is, with chemical compounds that can react with polymers inside the extrusion barrel. For instance, Harada et al. [26] studied the addition of different isocyanates in a PLA/PCL blend and observed that there was a significant reduction in the size of the PCL drops. In addition, Semba et al. [27], studied the addition of a dicumyl peroxide in a PLA/PCL blend and observed the same behavior.

In this work, a biobased TPE (polyurethane nature) was used as an elastomeric phase to improve the properties of PHB through melt blending. This strategy has been applied in previous works of our group [24,28,29] and those previous experiences have taught us that the interaction with PHAs is not very strong, resulting in low toughness enhancements and poor tensile strength. An attempt was made to improve the interaction of blends containing poly(hydroxybutyrate-co-valerate) (PHBV), cellulose, and a thermoplastic polyurethane (TPU) by adding reactive agents to act as compatibilizers [29]. The promising results obtained in that work led us to replicate the strategy with PHB and a biobased TPE, expecting similar results in terms of compatibilization of both polymers.

**Figure 1:**
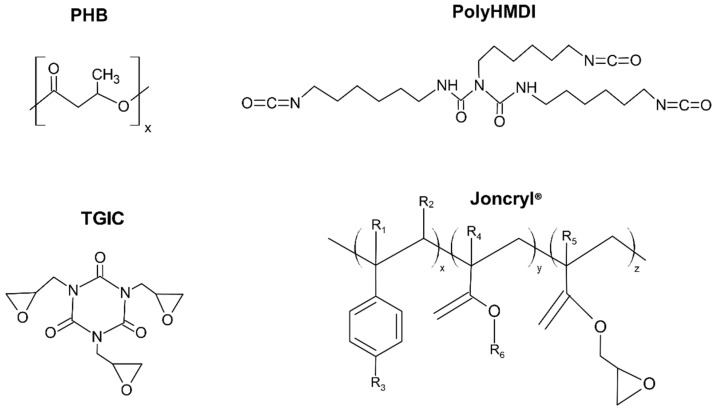
Chemical structures of materials used in this study.

Three reactive agents with different functional groups, poly-hexametylene diisocianate (polyHMDI), Joncryl^®^ ADR-4368 (a commercial multi-epoxy-functionalized styrene-acrylic oligomer), and triglycidyl isocyanurate (TGIC), depicted in Figure 1, were chosen to find out which one is more effective in this new system. Epoxy, isocyanates, and isocyanurates groups can potentially react with alcohol and carboxylic acid groups, which are present at both polyester and polyurethane chain ends [30].

To assess the influence of the TPE content and the reactive agents, full characterization of the blends was performed, including the influence of the orientation during processing and aging. Additionally, the processing ability of the blends in thermoforming was analyzed using a novel procedure developed by us in a pilot plant scale thermoforming station.

## 2. Materials and Methods

### 2.1. Materials

Poly(3-hydroxybutyrate) (PHB) was purchased from BIOMER (Krailling, Germany) in pellet form (P309-E). A biodegradable thermoplastic elastomer (TPE) NPEL208 was supplied by the NaturePlast (Ifs, France). Two of the three reactive agents used, triglycidyl isocyanurate (TGIC) and poly-hexamethylene diisocyanate (PolyHMDI), were supplied by Sigma-Aldrich (part of Merck KGaA, Darmstand, Germany). The last reactive agent, Joncryl^®^ ADR 4368-C, was supplied by BASF (Ludwigshafen, Germany).

### 2.2. Blend Preparation

The PHB and TPE used in this study were dried at 60 °C for at least 2 h, before being used, in a Piovan DPA 10 (Santa Maria di Sala, Italy), while the compatibilizers (TGIC, PolyHMDI, and Joncryl^®^) were used as received.

The PHB and PHB/TPE blends, with different amount of each one of them, and reactive agents (TGIC, PolyHMDI and Joncryl^®^) were obtained by mixing pellets of both polymers and the compatibilizer in a single-screw extruder equipped with a screw Maddock and L/D ratio = 25 (Rheomix 3000P ThermoHaake, Karlsruhe, Germany). The flat nozzle was coupled with calendrer to obtain sheets of 400 µm nominal thickness.

The temperature profile in the extruder used from hopper to nozzle was 140/150/175/175 °C and the rotation speed was 140 rpm. Samples of neat PHB were processed under identical conditions as the blends. All the components were manually premixed before extrusion and fed to the main hopper by the extruder feeder.

Two PHB/TPE compositions were developed, one with 15% and the other with 30% of TPE. From these compositions the effect of the compatibilizers (TGIC, PolyHMDI and Joncryl^®^) were tested.

The nomenclature used for naming the blends is as follows: PHB/15TPE and PHB/30TPE for the blend system without compatibilizer, and PHB/15TPE-polyHMDI, PHB/15TPE-TGIC, PHB/15TPE-Joncryl, PHB/30TPE-polyHMDI, PHB/30TPE-TGIC, and PHB/30TPE-Joncry for the compatibilized blends. Table 1 summarizes the samples studied and the relative compositions.

### 2.3. Characterization

#### 2.3.1. Thermoforming Ability

Thermoformability can be defined as the ability of a material to be successfully thermoformed into a sample with the shape of the mold. Such thermoformed shape must be reproducible and must have a controlled thickness distribution. In order to assess the thermoformability of the polymer blends and to determine which is the thermoforming temperature range, we developed a procedure based on testing different process conditions, making a visual inspection of the obtained samples and comparison with the original mold [31].

**Figure 2:**
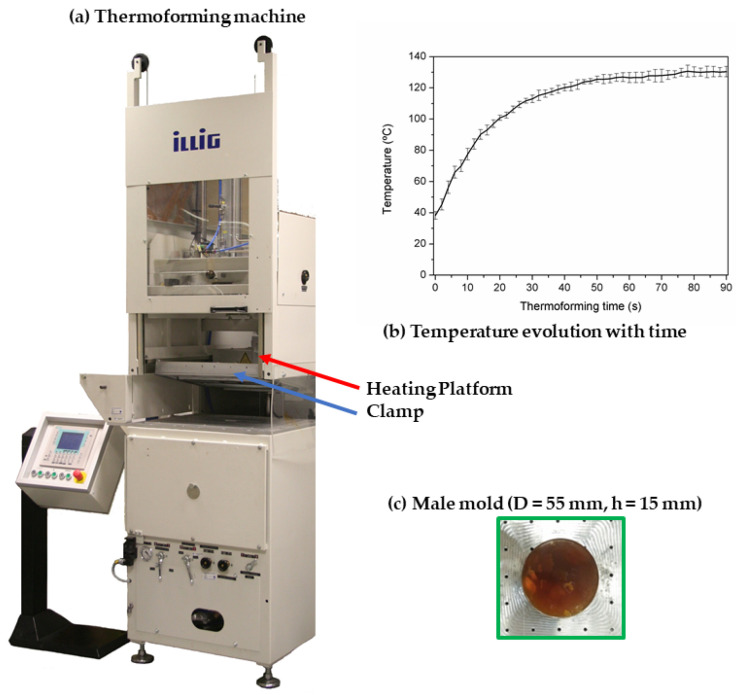
Image of the (**a**) thermoforming facility; (**b**) evolution of temperature with time at this processing conditions; and (**c**) male mold.

The thermoforming ability test was conducted in a pilot plant (SB 53c, Illig, Helmut Roegele, Germany) equipped with an infrared emitter heating device consisting in 15 long waves infrared emitters (see Figure 2a). This heating device was mounted under a sliding platform coupled with a clamp, (Figure 2b). The platform stayed 21 cm above the base where the film stayed during the heating step. After the heating time, the heater platform slid back, and the male mold (Figure 2c) raised under the heated film. Then, vacuum was applied between the film and the base, which led to the reproduction of the mold shape. In all the experiments, the heater was set to 600 °C, while the heating time was changed in order to control the temperature of the polymer sheet [32]. The surface temperature at different locations was measured (both in the upper and the lower surface) as a function of heating time and obtained a relation between the heating time and the sheet temperature (as shown in Figure 2b). The temperature of the sheet followed a logarithmic trend with respect to the heating time. By controlling this time, the temperature of the sheet could be selected. This temperature reached a plateau at about 50 s with this setup (around 130 °C), which was high enough to soften the PHB. The mold used was a cylindrical male of 55 × 15 mm (diameter × depth) (see Figure 2c).

A square grid pattern (1 × 1 cm) was printed on the sheets, in order to follow the deformations occurring during molding. Visual inspections of the thermoformed structures (trays) were performed to assess the thermoforming ability of the compositions. Photographs were taken for the record.

**Figure 3:**
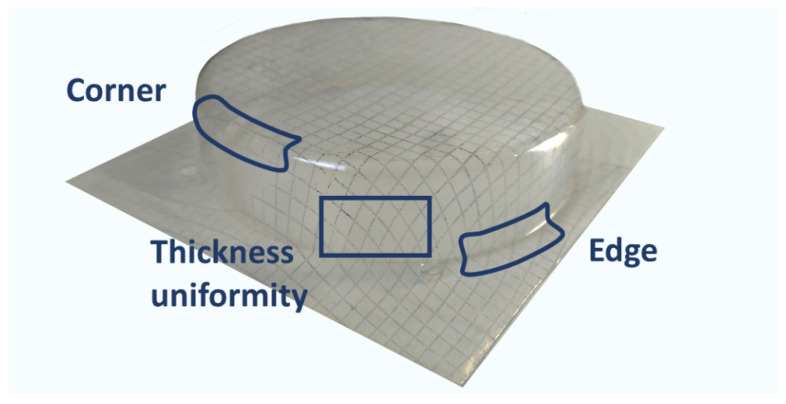
Picture of a thermoformed specimen. It shows the areas that are examined in order to evaluate the quality of the thermoforming.

After the thermoforming process, with the aid of the deformed grid, three parameters related to the mold shape reproducibility and the thickness distribution of the molded specimen were assessed. These three parameters (Figure 3) are described as follows:

(a) Edge inspection: Assesses the linearity in the joint section between the flat surface of the original sheet and the onset of the deformation (for the case of a tray-shaped mold, this would be the line defined by the intersecting planes of the original flat sheet and the vertical sides of the tray).
(b) Corner inspection: Provides information about the mold reproducibility at the corners of the tray.
(c) Thickness inspection: Evaluates the uniformity in the path and span of the squares in the grid (the shape of the grid elements is related to the local draw ratio and to the thickness distribution; high draw ratios result in high span and low thickness. On the other hand, even square-grid deformation is related to a uniform thickness distribution).

Each parameter was classified as “bad” (red color, cross sign), “intermediate” (blue color, wave sign), or “good” (green color, tick mark). The overall behaviour of the thermoforming conditions is set upon the evaluation of the three parameters represented in Figure 3. If any of them is considered as bad, then the thermoforming conditions are bad. If all the three parameters are good, then the conditions are good. When there is a combination between intermediate and good, then it is classified as intermediate. This procedure was used in this case to establish the thermoforming temperature range for each composition. Hence, the wider the temperature range, the higher the thermoforming ability of the composition.

#### 2.3.2. Mechanical Characterization

The mechanical characterization of the samples was performed by tensile tests according to the ASTM D638 standard. Dumb-bell samples were die-cut from the films in both MD (machine direction) and TD (transversal direction). Tests were carried out in a universal testing machine (Shimadzu AGS-X 5000N, Kyoto, Japan) equipped with a 500 N load cell at room temperature at a crosshead rate of 10 mm/min. The samples were tested immediately after being processed (0 days) and after 15 days of aging, to explore the effect of secondary crystallization on their mechanical performance.

**Figure 4:**
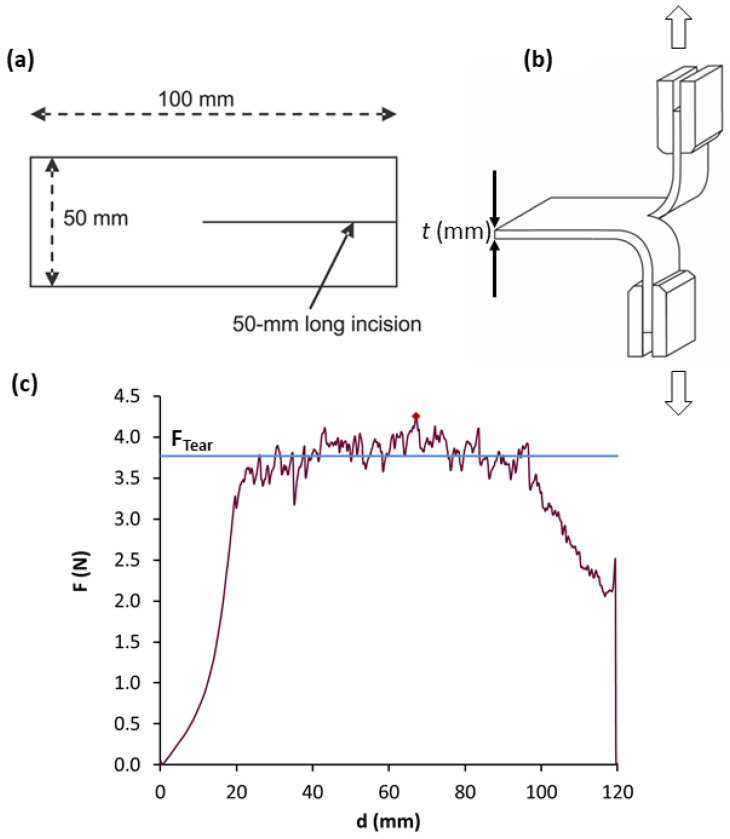
Sketch of the specimen (**a**), tear test direction (**b**) and determination of tear strength as the average tear force (F_Tear_) per thickness (*t*) unit (**c**).

Tear tests were also performed in MD and TD using the same equipment according to UNE-EN ISO 6383-1/200 standard, at 200 mm/min until their fracture. From the corresponding force vs. displacement curves, the tear strengths were calculated, as the average tear force per thickness unit (depicted in Figure 4). As well as in the case of tensile tests, the samples were tested at 0 and 15 days of aging.

#### 2.3.3. Morphology Characterization

The morphology of neat PHB and PHB/TPE blends, with and without compatibilizers (TGIC, PolyHMDI and Joncryl^®^), was assessed by scanning electron microscopy (SEM) using a high-resolution field-emission JEOL 7001F microscope (Tokyo, Japan). The samples were fractured in liquid nitrogen and were covered by sputtering with a thin layer of Pt, prior to SEM observation. From selected representative SEM images (at 2500× magnification), the diameters of the droplets corresponding to the dispersed phase were measured using Fiji^®^ software [31].

#### 2.3.4. Differential Scanning Calorimetry (DSC)

DSC experiments were conducted on a DSC2 (Mettler Toledo, Columbus, OH, USA), equipped with an intracooler (Julabo FT900, Seelbach, Germany) calibrated with an Indium standard before use. The weight of the DSC samples was around 5 mg. Samples were first heated from −20 °C to 190 °C at 10 °C/min and kept for 1 min at 190 °C, then cooled to −20 °C at 10 °C/min, kept for 1 min at −20 °C, and finally heated to 190 °C at 10 °C/min. Melting temperatures (*T_m_*) and enthalpies (Δ*H_m_*), as well as crystallization temperatures (*T_c_*) and enthalpies (Δ*H_c_*), were calculated from the second heating and cooling curves, respectively. Crystallinity (*X_c_*) of the PHB phase in the blends was determined by applying the following expression:

(1) $$X_{c}\left(\%\right)=\frac{\Delta H_{m}}{\Delta H_{m}^{0}\times w_{PHB}}\times 100\;$$

where Δ*H_m_* (J/g) is the melting enthalpy of the polymer matrix, Δ*H*^0^*_m_* is the melting enthalpy of 100% crystalline PHB (146 J/g) [33], and *w_PHB_* is the polymer weight fraction of PHB in the blend.

#### 2.3.5. Thermogravimetry with Coupled Fourier Transformed Infrared (TGA-FTIR)

TGA-FTIR analysis were performed using a TG 209 F1 Libra^®^ (Netzsch, Selb, Germany). The samples were heated from 30 to 620 °C, at a heating rate of 10 °C /min under nitrogen flow. The characteristic temperatures, T_5%_ and T_d_, corresponded, respectively, to the initial decomposition temperature (5% weight loss) and to the maximum degradation rate temperature, measured as the maximum in the curve corresponding to the first derivative of the thermogravimetric analysis (DTG). Fourier transformed infrared spectroscopy (FTIR) analyses from the volatile compounds generated during the heating ramp were carried out on ALPHA II (Bruker, Billerica, MA, USA), and the data were analyzed using OPUS software.

## 3. Results

### 3.1. Characterization

#### 3.1.1. Thermoforming Ability

**Figure 5:**
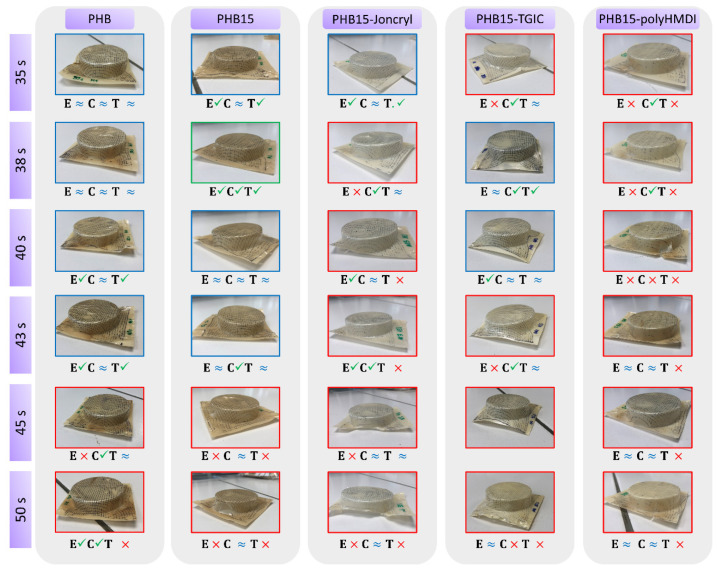
Photographs and assessment of thermoforming ability of the trays, showing different heating times for Neat PHB, PHBV/15TPE blend, and the PHB/15TPE compatibilized blends. The frame color indicates the overall quality of thermoforming, based upon the inspection of edges (E), corners (C) and thickness uniformity (T).

**Figure 6:**
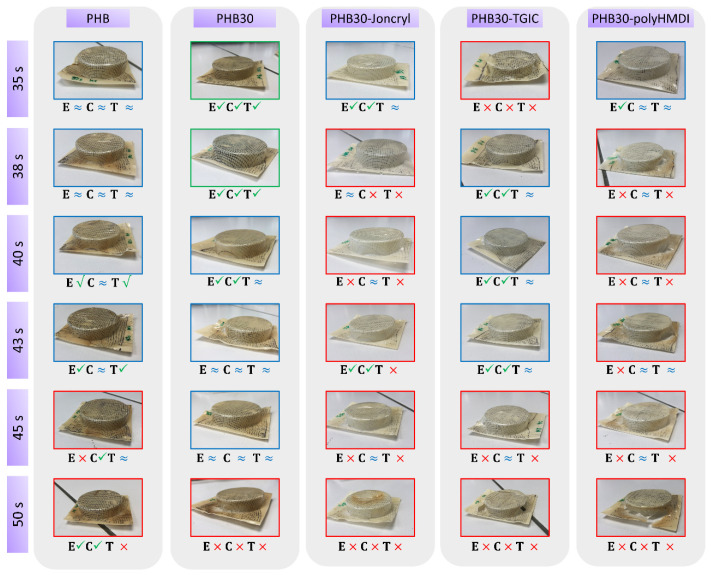
Photographs and assessment of thermoforming ability of the trays, showing different heating times for Neat PHB, PHB30 blend and the PHB30 compatibilized blends. The frame color indicates the overall quality of thermoforming, based upon the inspection of edges (E), corners (C) and thickness uniformity (T).

The evaluation of the thermoforming ability was carried out by means of visual inspection of the thermoformed trays and measurement of the thickness distribution, as described in the Materials and Methods section. Figure 5 and Figure 6 summarize the results of all the PHB/TPE blends studied (with and without the compatibilizers) as a function of the heating time in the thermoforming machine, including representative pictures of the thermoformed trays.

From Figure 5, it can be seen that the incorporation of 15 wt% TPE to PHB (without reactive agents) resulted in a slight improvement of the thermoforming ability with respect to neat PHB at 38 s of heating. However, from previous works [32], we expected to find out less thickness variation and a significant increase in the thermoforming time interval (i.e., increasing the processing temperature window where good trays could be produced). Only in the case where 30% TPE was added to PHB (Figure 6), a significant improvement of the thermoforming ability with respect to PHB, better thickness distribution, and an increase in the time frame (35 to 38 s of heating time) could be seen.

When the compatibilizers were added to the blends, not only did they not show enhancement with respect to the uncompatibilized blends, but the addition of Joncryl^®^ and HMDI decreased the thermoforming ability behavior with respect to neat PHB. In the case of the blends compatibilized with TGIC, the behavior improved with respect to neat PHB, but not as well as the uncompatibilized compositions.

As these results differ from the previous work where the addition of compatibilizers had a significant impact, improving the thermoforming ability of PHBV/Polylactic Acid (PLA) blends [32], further test and characterizations were carried out in order to elucidate this behavior.

#### 3.1.2. Mechanical Properties

##### Tensile Test

**Figure 7:**
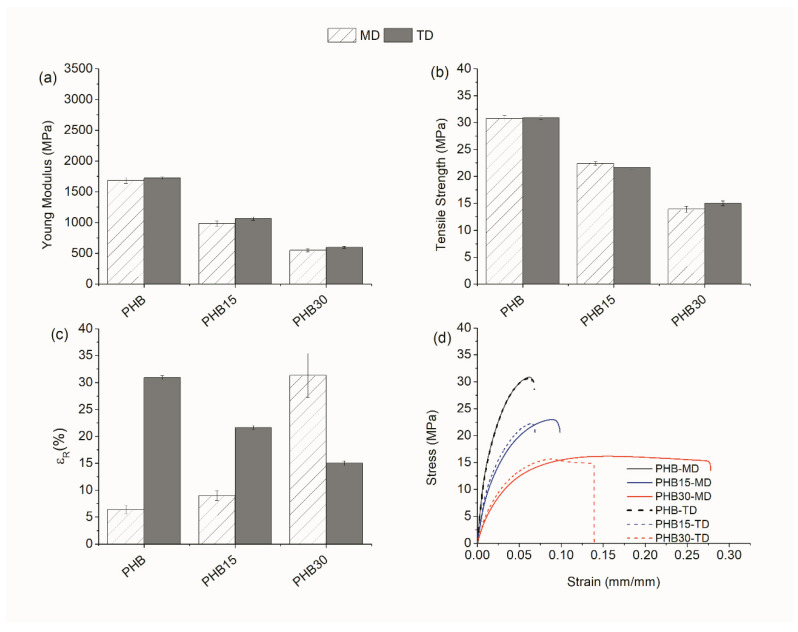
Mechanical properties at 0 days in MD and TD: (**a**) Modulus of elasticity, (**b**) tensile strength, (**c**) strain at break, and (**d**) representative stress vs. strain curves.

**Figure 8:**
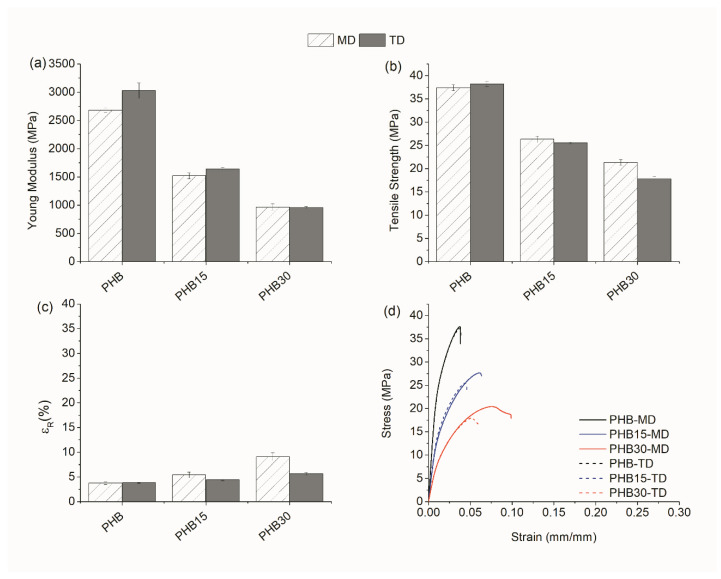
Mechanical properties at 15 days in MD and TD: (**a**) Modulus of elasticity, (**b**) tensile strength, (**c**) strain at break, and (**d**) representative stress vs. strain curves.

The mechanical properties of the samples in MD and TD were evaluated by uniaxial tensile tests up to break. Since PHB is known to show secondary crystallization phenomenon, which greatly affects its mechanical performance [33,34,35], tensile tests were carried on at 0 days and after 15 days of aging at room conditions. The modulus of elastic (E), tensile strength at yield (σ_max_), and elongation at break (ε_r_) of the pristine blends at 0 and 15 days of aging are represented in the bar diagrams (Figure 7 and Figure 8), respectively. In addition, representative stress–strain curves of PHB and the blends containing the TPE are depicted in Figure 7d and Figure 8d for clarification.

As evidenced in Figure 7a,b, the tensile performance of PHB/TPE blends without compatibilizer show that the addition of TPE decreases the mechanical strength and rigidity of the blends at 0 days, being more pronounced in those with a greater amount of TPE, in both MD and TD. With respect to elongation at the break, while the addition of TPE increases this value from 5 to 30%, in TD the trend is the opposite, finding a reduction from 30% to 10% (Figure 7c). After 15 days of aging (Figure 8), the values of E and σ_max_ increase in all cases. Secondary crystallization in PHB causes a raise of the tensile strength before yielding, which leads to brittle rupture. The addition of TPE decreases the tensile strength and rigidity of the samples with respect to neat PHB. Nevertheless, yielding of the values is not fully achieved before the rupture of the specimens and the values of ε_r_ were greatly reduced with respect to those at 0 days.

The addition of reactive agents to PHB/TPE blends was expected to improve the mechanical performance of the blends. Table 2 shows the parameters obtained from all the tensile tests, with and without reactive agents. Surprisingly, in most cases the performance of the blends with reactive agents were alike or even slightly worse than the ones without them.

For instance, when reactive agents are added to PHB15 blends, a slight decrease of the modulus of elasticity and tensile strength parameters in both MD and TD, at 0 or 15 days can be seen. Regarding the elongation at break (ε_r_), however, there is an exception when TGIC is used as compatibilizer, since in all cases it causes an increase in this parameter.

In PHB30 blends, at 0 days, the effect of adding reactive agents on the values of E and σ_max_ shows a moderate increase in MD and a decrease in TD, indicating some anisotropy induced by orientation. It is worthwhile noticing that the values of elongation at break in PHB30-TGIC follow the same trend as the ones with 15% TPE, being the only case where ε_r_ increases in both MD and TD. After 15 days of aging, the values of E and σ_max_ in the blends with 30% TPE increase, as expected from the secondary crystallization of PHB, with a reduction of the elongation at break.

In contrast to other studies carried out before, where the addition of compatibilizers showed favorable results in improving the mechanical properties [24], in this study the results reveal that the compatibilizers do not improve the interfacial adhesion of the TPE with the polymeric matrix.

##### Tear Strength

The mechanical performance of the films was also studied using tear testing, as this is a limiting property of PHB for its use in packaging. Hence, samples were tested in MD and TD, as well as their response at 0 days and 15 days of aging. Table 3 summarizes the parameters obtained from those tests.

Tear test results from pristine blends (PHB15 and PHB30) show that addition of 15% TPE to neat PHB caused a decrease of resistance to tear in MD respect to neat PHB at 0 and 15 days. The lower values of tensile strength and modulus of elasticity of the blends, discussed previously, agree with such a reduction on tear strength. However, when TPE content is 30%, in MD, the values of tensile strength do not further decrease with respect to PHB15. Indeed, in TD, there is a strong increase on the tear strength, when compared to the neat PHB, showing a very strong toughening effect.

The addition of the compatibilizers, in the PHB15 blends, slightly decreases the tear resistance in MD at 0 and 15 days. In TD, however, the trend is unclear, with a slight toughening effect in PHB15-Joncryl and PHB15-TGIC, but not in PHB15-polyHMDI. Regarding the PHB30 blends with reactive agents, the use of TGIC (and Poly-HMDI to a minor extent) seem to slightly improve the tear strength in MD and TD.

#### 3.1.3. Morphology Characterization

**Figure 9:**
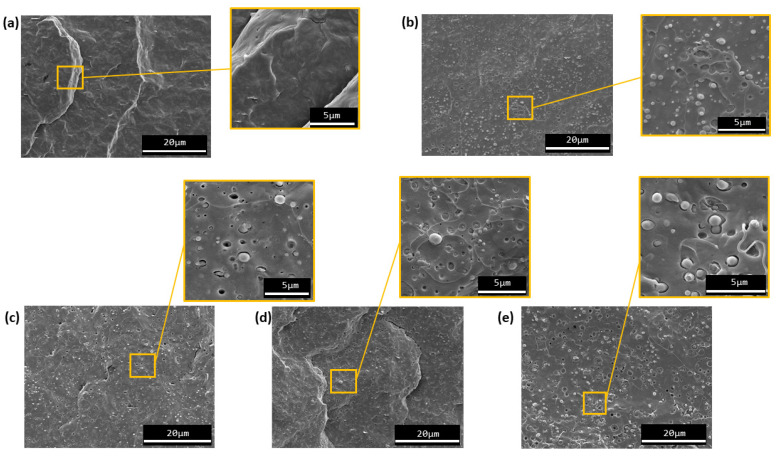
SEM micrographs of (**a**) Neat PHB, (**b**) PHB15, (**c**) PHB15-Joncryl^®^, (**d**) PHB15-TGIC, and (**e**) PHB15-polyHMDI.

**Figure 10:**
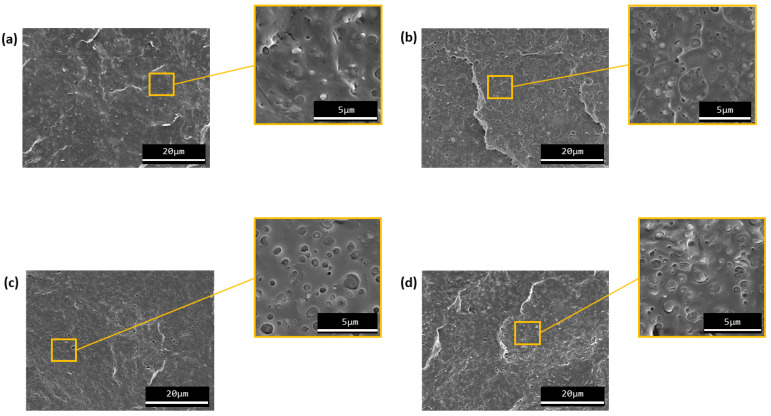
SEM micrographs of (**a**) PHB30, (**b**) PHB30-Joncryl^®^, (**c**) PHB30-TGIC, and (**d**) PHB30-polyHMDI.

The morphology of neat PHB and PHB/TPE blends with and without the compatibilizer was analyzed by SEM. Micrographs of PHB/5 and PHB30 without reactive agents and with 1 phr of polyHMDI, Joncryl^®^, and TGIC are depicted in Figure 9 and Figure 10, respectively.

The micrographs show that two phases (PHB and TPE) can be identified in the morphology of the blends. In all cases, the structure can be described as a typical drop-in matrix discrete phase, where TPE droplets are homogeneously dispersed in the PHB continuous matrix. This type of microstructure is evidence that the PHB/TPE blends prepared are immiscible. However, the small size of the dispersed phase domains indicates that there was a certain affinity between both polymers during the mixing stage. As the amount of TPE is increased, the size of the dispersed domains also increases, as seen when comparing Figure 9b and Figure 10a (PHB/15TPE and PHB/30TPE, respectively).

With the incorporation of the compatibilizers (polyHMDI, Joncryl^®^ and TGIC) the average TPE droplet size was not reduced (this can be notice by looking at Figure 9e and Figure 10d, for example). Only in the case of PHB30-TGIC, it seems that there is a slight decrease in the droplet size of the dispersed phase (Figure 10c), as well as lesser fraction of detached particles with respect to uncompatibilized PHB30. This may indicate a relative increase in compatibility between both phases.

SEM observations are in agreement with the mechanical properties, where there were no observed changes in PHB when the reactive agents were added as compatibilizers. It can be concluded, from this analysis, that the use of those compatibilizers did not produce any significant improvement in the PHB/TPE blends used in this study.

#### 3.1.4. Thermal Characterization

The thermal behavior of neat PHB and the prepared blends were studied by DSC and TGA. The DSC measurements were performed at 0 days after processing PHB and PHB/TPE films. The most important thermal parameters, obtained from heating/cooling scans after thermal history erasing, are summarized in Table 4. DSC curves can be found as Supplementary Material (Figure S1).

From the variation in melting and crystallization temperatures, as well as melting and crystallization enthalpies of PHB/TPE blends with respect to neat PHB (shown in Table 3), it can be seen that addition of TPE to PHB slightly hinders the crystallization, with lower melting temperatures and crystallinity indexes. These results are in agreement with the other works reported in literature about PHBV/TPU blends [24,28,36,37]. This phenomenon is reasonable considering the inter-molecular interactions between the phases in the liquid state. With both phases being partially compatible (not fully segregated), some entanglements between both polymers are plausible. As a consequence, the crystallization of the PHB from the melt is hampered and so the overall crystallinity is lower, and the crystal lamellae are thinner. However, once PHB crystallization takes places, the TPE phase is excluded from the crystals, giving a final completely segregated morphology, as seen in SEM micrographs of the previous section.

The addition of the reactive agents to PHB/TPE blends practically did not alter either the melting/crystallization temperatures or the overall crystallinity, compared to the blends without compatibilizers. These findings indicate that those chain extenders did not react directly with the PHB matrix.

**Figure 11:**
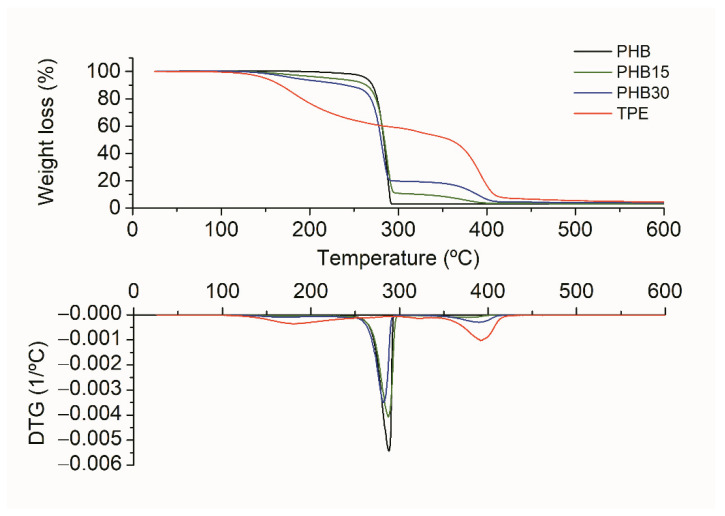
TGA and DTG curves of neat PHB, TPE, and PHB15 and PHB30 blends.

Thermogravimetric analysis of the blends can provide an explanation for these findings. In Figure 11 the TGA curves of PHB, TPE, PHB15, and PHB30 are shown. The onset degradation temperature (T_5%,_) and the maximum degradation temperature (T_d_) are summarized in Table 5.

Thermal degradation of PHB takes place suddenly, with an onset temperature at 264 °C and maximum degradation temperature at 288 °C [38], in a single weight-loss step. Regarding the bio-TPE, the derivative of weight loss vs. temperature (DGT curves in Figure 11) presents three peaks at 180 °C, 320 °C, and 390 °C. The first peak (ranging from 150 to 250 °C) could be attributed to mass loss of volatile compounds, such as plasticizers, that can be possibly contained within the TPE matrix. The peak at 320 °C corresponds with the first steps of polyurethane degradation, ascribed to urethane dissociation to form isocyanate and alcohol (depending on the composition, rupture of unstable side chains may also occur). The second stage, that takes place between 300 and 420 °C, is linked with the degradation of the soft segments [39]. The low ratio between the peaks at 320 °C and 390 °C agrees with a soft elastomer, with higher content of soft segments.

**Figure 12:**
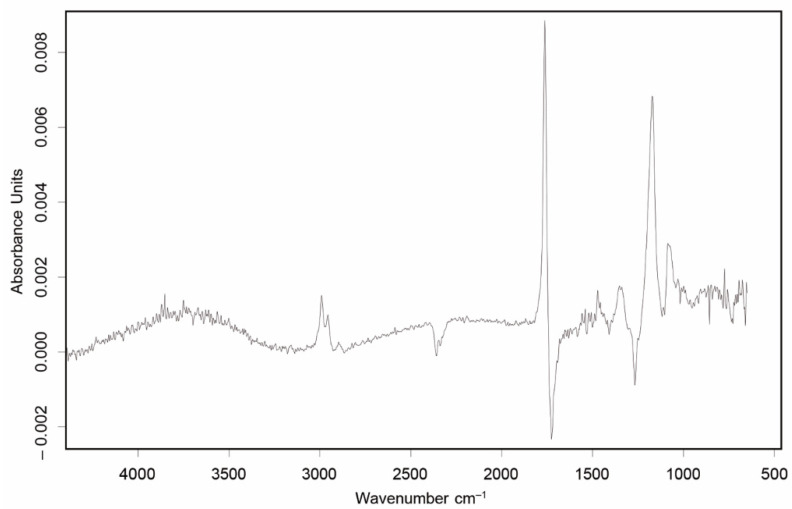
IR spectrum from TPE-TGA gases at 187 °C.

FTIR from volatiles during TGA analysis of TPE was conducted (spectra available in Figure S2) to reveal the nature of the compounds released. During the first peak at 180 °C, IR spectra were analyzed at four temperatures, 176, 187, 208, and 220 °C, to be sure that all volatiles of that range were of the same nature. Figure 12 shows FT-IR spectrum obtained at 187 °C, as an example, since similar spectra and results were obtained for the temperatures analyzed. The possible presence of -OH groups (absorption band at 3700 cm^−1^), C-H extension bands (around 2990 and 2958 cm^−1^), C=O stretching band of esters (1767 cm^−1^), and C-O stretching band of esters (at 1170 cm^−1^) can be observed [40].

**Figure 13:**
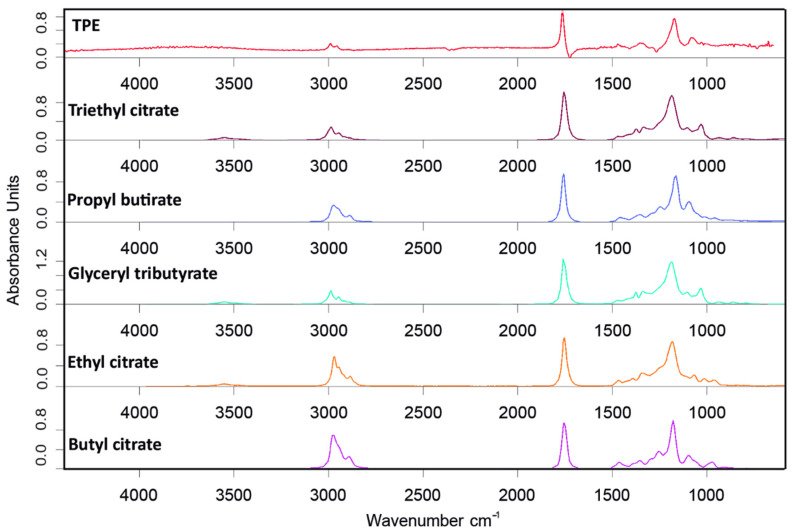
FTIR from TPE degradation at 187 °C and possible compounds that match with the spectra.

Using the OPUS software database, it was possible to relate the spectrum obtained in the degradation of TPE with other spectra that present the same functional groups. Figure 13 presents the outcome from such analysis, showing good agreement with the spectra of glyceryl tributyrate, tributyl citrate, and butyl citrate. As citrates are typically group that are used as “eco-friendly” plasticizers [40,41,42], it is most likely that they were in this bio-TPE to either facilitate the processing and/or tunning the properties of the elastomer. It is worthwhile to highlight that according to the TGA, c.a. 40% wt. of the bio-TPE corresponds to plasticizers and/or volatile compounds.

PHB/TPE blends, as expected, showed the degradation stages of TPE and PHB. Figure 10 shows that for both PHB15 and PHB30 there is a DGT peak around 280 °C, ascribed to the degradation of PHB, and a second peak at 390 °C, related to the degradation of the TPE. However, the apparent onset degradation temperatures of the blends with 15 and 30 wt% TPE content are considerably lower than neat PHB.

Considering the analysis of the TGA performed to TPE, the lower values of T_5%_ of the blends can be explained by the higher content of volatile plasticizers present in TPE, which would represent c.a. 6 and 12% in weight for PHB15 and PHB30, respectively. The maximum degradation temperatures of the blends, listed in Table 4 are comparable to those corresponding to neat PHB. These values indicate that the presence of TPE does not affect the thermal stability of PHB, in concordance with what has been previously reported [24].

Just like PHB/TPE blends without compatibilizer, the PHB/TPE blends with compatibilizers showed the same degradation peaks. As previously discussed, the onset degradation temperatures of the blends are affected by the presence of volatile compounds from TPE. Regarding the maximum degradation temperatures, the samples with compatibilizers were similar to those corresponding with PHB15 and PHB30. There is an exception, however, in the blends with polyHMDI, where T_dmax_ are lower than PHB and the other blends with 15 and 30 wt% TPE content, indicating that the use of this reactive agent lowers the thermal resistance of the blends.

## 4. Discussion

In this study a biodegradable elastomer (TPE) was used in order to improve the properties of neat PHB. Through SEM, it was observed that PHB/TPE blend are immiscible, although the present a two-phase morphology microstructure in the form “drop-in matrix”. These blends show small size TPE particles between 0.76–0.49 µm, similar to blends with TPU [24,29], and a good dispersion of these in the matrix, which means certain affinity between the phases. In order to improve the miscibility of the blends, three reactive agents commonly used in reactive extrusion were added as compatibilizers (polyHMDI, Joncryl^®^ ADR4368-C, and TGIC). They are known to react with -OH chain ends of polymers and they were added in a concentration of 1 phr, which was proven sufficient in similar previous systems [29,32].

The incorporation of TPE in PHB led to an initial reduction on the tensile modulus of elasticity and tensile strength, but an enhancement in the elongation at break, with an overall increase in static toughness. Remarkable differences were obtained when the amount of TPE was higher. All this has been attributed to the toughening effect of the TPE on the PHB matrix. However, after 15 days of aging, the tensile properties evolved towards higher stiffness and strength, with a considerable reduction on elongation at break. This behavior is in agreement with the common phenomenon of secondary crystallization of PHB, limiting the toughening effect of the elastomeric phase. Regarding thermoforming, the addition of TPE, both 15 and 30 wt.%, slightly improved the processing window with respect to neat PHB. These results indicate that the addition of TPE to neat PHB overall improves its industrial applicability. However, contrary to expectations, the addition of the compatibilizers to the blends gave similar results to the PHB/TPE blends without it, despite of a slight enhancement in the thermoforming capacity of PHB when TGIC was incorporated to the blend. This lack of compatibility improvement was evidenced in SEM analysis. Surprisingly, there was not any variation on the morphology of the bends with the addition of compatibilizer.

Similarly, in DSC experiments and it was common to all three reactive agents used, based either on epoxy (Joncryl^®^ and TGIC) or isocyanate (HMDI) reactive species. TGA results have shown a degradation peak in TPE that can be related with the degradation/volatilization of plasticizers present in TPE, which were present in a 40% weight. FTIR from volatiles confirmed that those plasticizers can easily be citrates, which are known to be used as plasticizers.

Hence, it is reasonable for us to assume that these plasticizers, which have -OH groups and are smaller in size (more labile) than polymer end chains, react with compatibilizers faster than the polymer chains, preventing their functionality. This explains why the effect of the reactive agents was barely noticed in the blends. It also provides an explanation to why TGIC was the only reactive agent that slightly altered the properties, since it has the lowest molecular weight of all reactive agents. Therefore, the activity of TGIC (number of functional groups per unit weight) was the highest, and it could be more likely than Joncryl ^®^ and polyHMDI to react with some polymer chain ends.

In this type of situation, a better strategy would probably be to perform the blend with the polymer matrixes without plasticizers first, and afterwards add the additives necessary to tailor the properties of the plastic.

## 5. Conclusions

The development of environmentally sustainable functional plastics requires the development of materials with combined properties, such as mechanical strength, toughness, and industrial processability. The addition of reactive agents in order to make the rigid matrix of PHB compatible with an elastomeric phase (TPE), even though it may seem a reasonable approach, was not able to significantly improve the properties of these blends, regardless of the nature of the reactive agents.

The reactive extrusion modification strategy was proven to be efficient in numerous studies in order to increase the compatibility of phases such as those presented in this work. However, the presence of plasticizers in the TPE and their competition in reactivity with the reactive agents (TGIC, Joncryl^®^ and polyHMDI) neutralized their effect on the compatibilization of both phases, and did not enhance the properties of the blends.

This consideration is of relevance in industrial environments, where additive-free polymer grades are not available, or where the properties of the base polymers are strongly influenced by additives.

## Figures and Tables

**Table 1:** List of compounds and their composition.

Sample	Neat PHB (wt%)	TPE (wt%)	TGIC (phr) ^1^	PolyHMDI	Joncryl^®^
PHB	100	-	-	-	-
PHB15	85	15	-	-	-
PHB15-TGIC	85	15	1	-	-
PHB15-Joncry^®^	85	15	-	-	1
PHB15-PolyHMDI	85	15	-	1	-
PHB30	70	30	-	-	-
PHB30-TGIC	70	30	1	-	-
PHB30-Joncry^®^	70	30	-	-	1
PHB30-PolyHMDI	70	30	-	1	-

^1^ phr (per hundred resin) refers to 100 weight units of PHB/TPE altogether.

**Table 2:** Tensile test parameters of the blends with the addition of the reactive agents, at 0 and 15 days of aging.

		0 Days			15 Days		
Samples		E (MPa)	σ_max_ (MPa)	ε_R_ (%)	E (MPa)	σ_max_ (MPa)	ε_R_ (%)
PHB	MD	1680	30.8	6.4	2680	37.4	3.8
	TD	1720	30.9	6.7	3030	38.2	3.8
PHB15	MD	990	22.4	9.0	1520	26.3	5.4
	TD	1070	21.6	6.6	1640	25.5	4.4
PHB15-TGIC	MD	820	21.1	11.1	1290	25.4	6.8
	TD	760	18.1	10.0	1260	22.0	5.4
PHB15-Joncryl^®^	MD	920	20.5	7.6	1450	26.1	5.1
	TD	1050	20.2	6.0	1630	25.0	4.1
PHB15-polyHMDI	MD	960	20.4	8.1	1430	24.8	5.3
	TD	1070	20.4	5.1	1570	23.3	3.9
PHB30	MD	550	13.9	31.3	970	21.3	9.1
	TD	600	15.0	11.7	960	17.8	5.7
PHB30-TGIC	MD	610	17.9	31.8	910	20.7	9.3
	TD	560	15.1	14.7	860	16.9	6.6
PHB30-Joncryl^®^	MD	660	16.6	13.9	880	19.8	9.3
	TD	600	13.3	7.5	750	14.3	5.5
PHB30-polyHMDI	MD	630	17.6	17.4	1010	21.5	7.4
	TD	510	14.4	11.0	1080	20.0	5.8

**Table 3:** Tear strength parameters obtained for the blends at 0 and at 15 days of aging.

		0 Days	15 Days
Samples		Tear Strength (N/mm)	Tear Strength (N/mm)
PHB	MD	10.1	8.8
	TD	10.8	9.5 *
PHB15	MD	7.2	4.8
	TD	8.6	6.3
PHB15-TGIC	MD	6.6	5
	TD	10.9 *	9.5 *
PHB15-Joncryl^®^	MD	5.5	3.7
	TD	8.9	7.5 *
PHB15-polyHMDI	MD	6.9	3.9
	TD	7.2	5.9 *
PHB30TPE	MD	7.5	4.4
	TD	17.7 *	14.6 *
PHB30-TGIC	MD	7.7	5.5
	TD	29.2 *	13 *
PHB30-Joncryl^®^	MD	2.6	1.9
	TD	8.7 *	4.9 *
PHB30-polyHMDI	MD	8.8	3.9
	TD	26.5 *	8.7 *

* Indicates that samples showed fracture deviation during test.

**Table 4:** DSC data of PHB, TPE, and PHB/TPE 15 and 30 wt% with and without compatibilizers blends.

Composition	*T_c_* (°C)	Δ*H_c_* (J/g)	*T_m_*(°C)	Δ*H_m_* (J/g)	*X_c_* (%)
PHB	122	90	172	93	64
TPE	67	-	19/99 ^1^	-	-
PHB/15TPE	108	64	168	66	53
PHB/15TPE-TGIC	108	59	167	64	52
PHB/15TPE-Joncryl^®^	109	72	169	73	59
PHB/15TPE-polyHMDI	108	70	169	69	56
PHB/30TPE	104	53	165	57	55
PHB/30TPE-TGIC	106	61	167	62	61
PHB/30TPE-Joncryl^®^	106	59	166	62	61
PHB/30TPE-polyHMDI	107	61	167	61	60

^1^ Two melting peaks.

**Table 5:** TGA data of PHB, TPE, and PHB/TPE 15 and 30 wt% with and without compatibilizers blends.

Composition	T_5%_ (°C)	T_dmax_ (°C)
PHB	264	288
PHB/15TPE	224	287
PHB/15TPE-TGIC	214	283
PHB/15TPE-Joncryl^®^	230	286
PHB/15TPE-polyHMDI	220	265
PHB/30TPE	183	281
PHB/30TPE-TGIC	193	282
PHB/30TPE-Joncryl^®^	191	285
PHB/30TPE-polyHMDI	185	274
TPE	150	392

## Data Availability

The data presented in this study are available on request from the corresponding author.

## Supplementary Materials

The following are available online at https://www.mdpi.com/article/10.3390/ma15031226/s1, Figure S1: DSC Curves, Figure S2: TGA/FTIR Curves TPE.
