# Supplementary material for: Role of Plasticizers on PHB/bio-TPE Blends Compatibilized by Reactive Extrusion
Source: Materials (Basel). 2022 Feb 7;15(3):1226. doi: 10.3390/ma15031226 (PMC8840646; doi:10.3390/ma15031226)

materials

MDPI

Supplementary Materials.

Role of plasticizers on PHB/bio-TPE blends compatibilized by reactive extrusion.

Kerly Samaniego[1], Armando Matos[1], Estefanía Sánchez-Safont[1], María V. Candal[2], Jose M. Lagaron[3], Luis Cabedo[1] and Jose Gamez-Perez[1,*].

[1] Polymers and Advanced Materials Group (PIMA), Universitat Jaume I, 12071 Castelló de la Plana, Spain; samanieg@uji.es (K.S.); amatos@uji.es (A.M.); esafont@uji.es (E.S.-S.); lcabedo@uji.es (L.C.)
[2] Technology and Science Group, Valencian International University (VIU), 46002 Valencia, Spain; mariavirginiacandal@campusviu.es
[3] Novel Materials and Nanotechnology Group, Institute of Agrochemistry and Food Technology (IATA), Spanish National Research Council (CSIC), 46980 Valencia, Spain; lagaron@iata.csic.es * Correspondence: gamez@uji.es

Figure S1. DSC Curves.

(A) PHB/TPE 15%

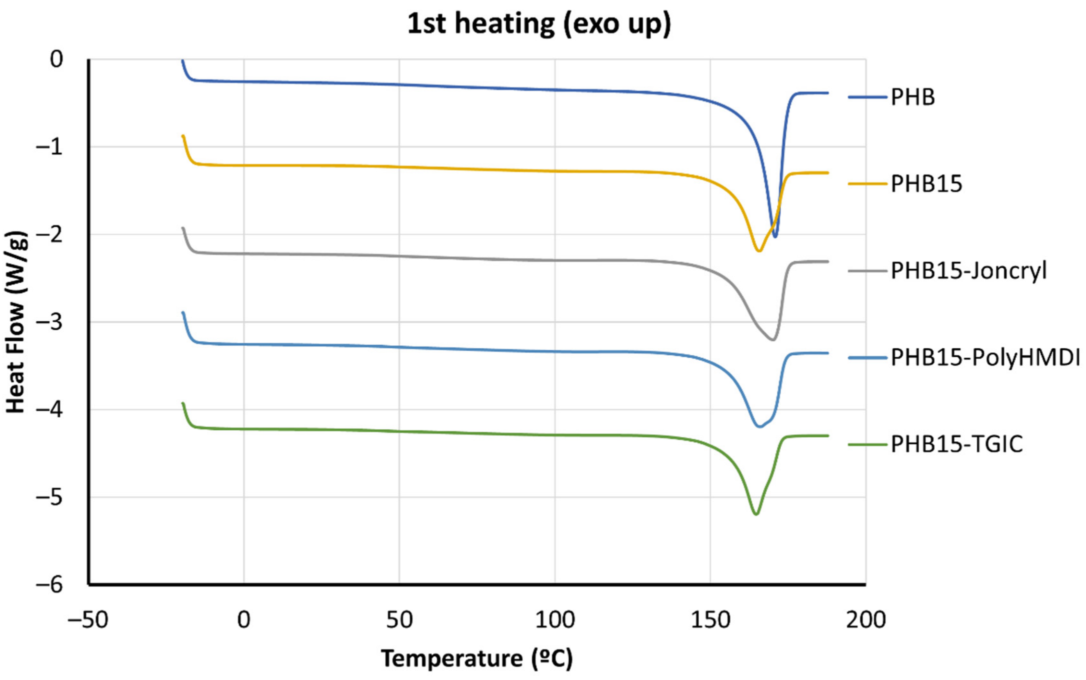

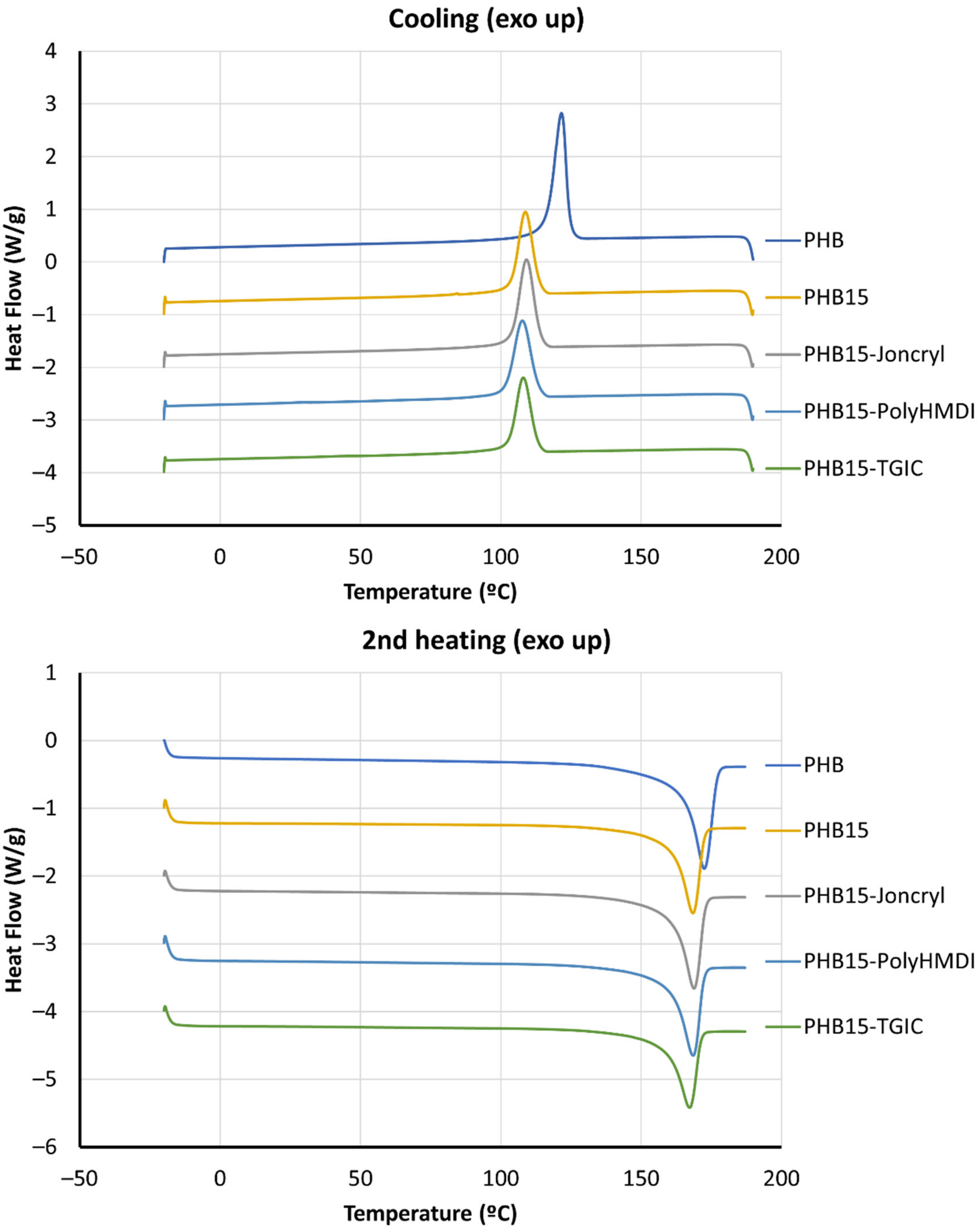
Cooling (exo up)
Heat Flow (W/g)
Temperature (ºC)
PHB
PHB15
PHB15-Joncryl
PHB15-PolyHMDI
PHB15-TGIC
2nd heating (exo up)
Heat Flow (W/g)
Temperature (ºC)
PHB
PHB15
PHB15-Joncryl
PHB15-PolyHMDI
PHB15-TGIC

(B) PHB/TPE 30%

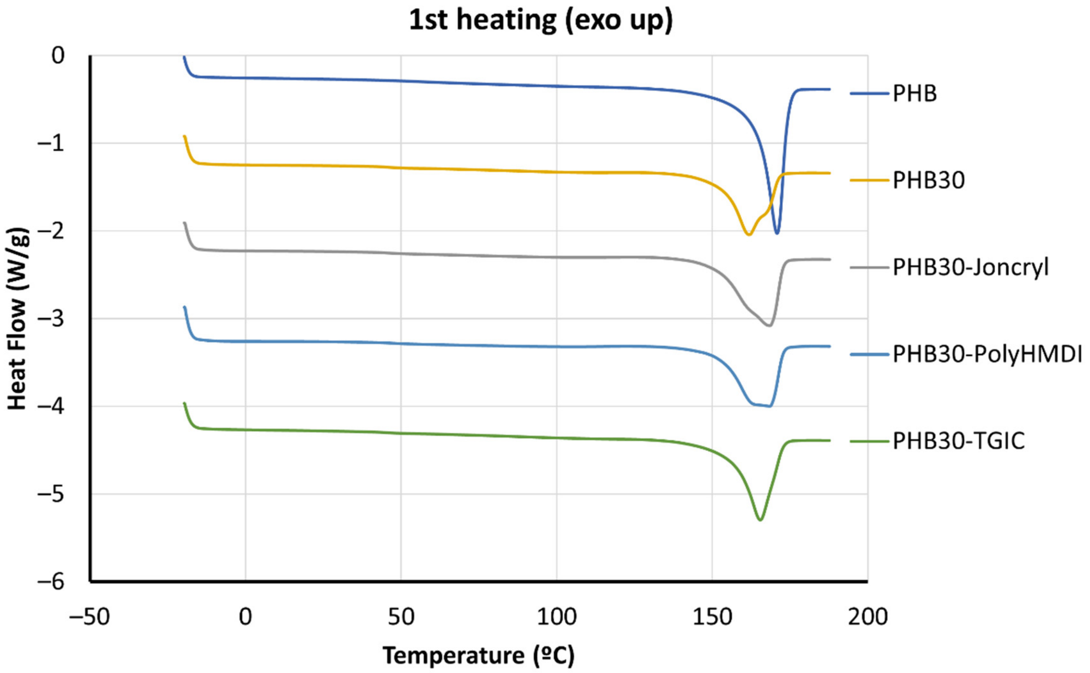
1st heating (exo up)
Heat Flow (W/g)
0
−1
−2
−3
−4
−5
−6
−50
0
50
100
150
200
Temperature (ºC)
PHB
PHB30
PHB30-Joncryl
PHB30-PolyHMDI
PHB30-TGIC

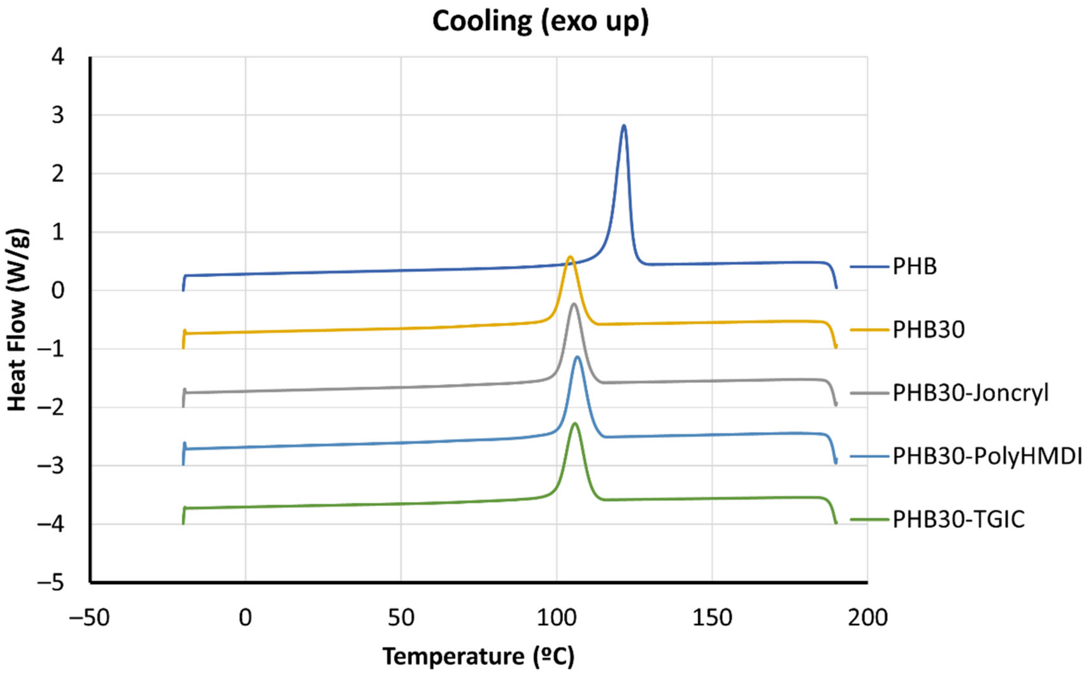
Cooling (exo up)
Heat Flow (W/g)
4
3
2
1
0
−1
−2
−3
−4
−5
−50
0
50
100
150
200
Temperature (ºC)
PHB
PHB30
PHB30-Joncryl
PHB30-PolyHMDI
PHB30-TGIC

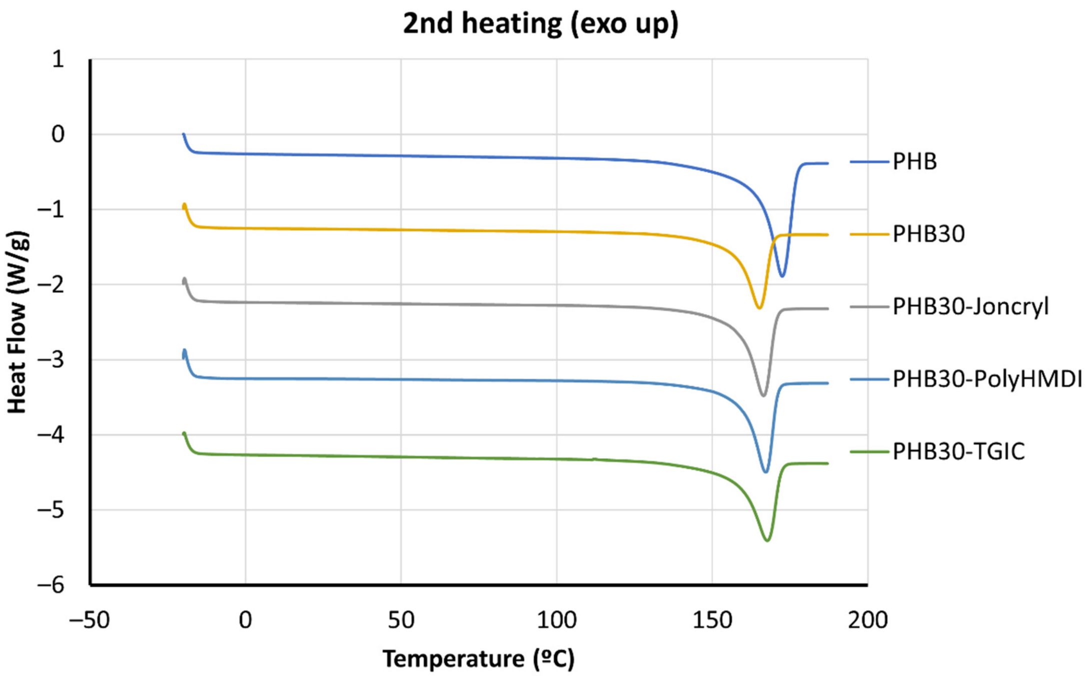

Figure S2. TGA/FTIR Curves TPE

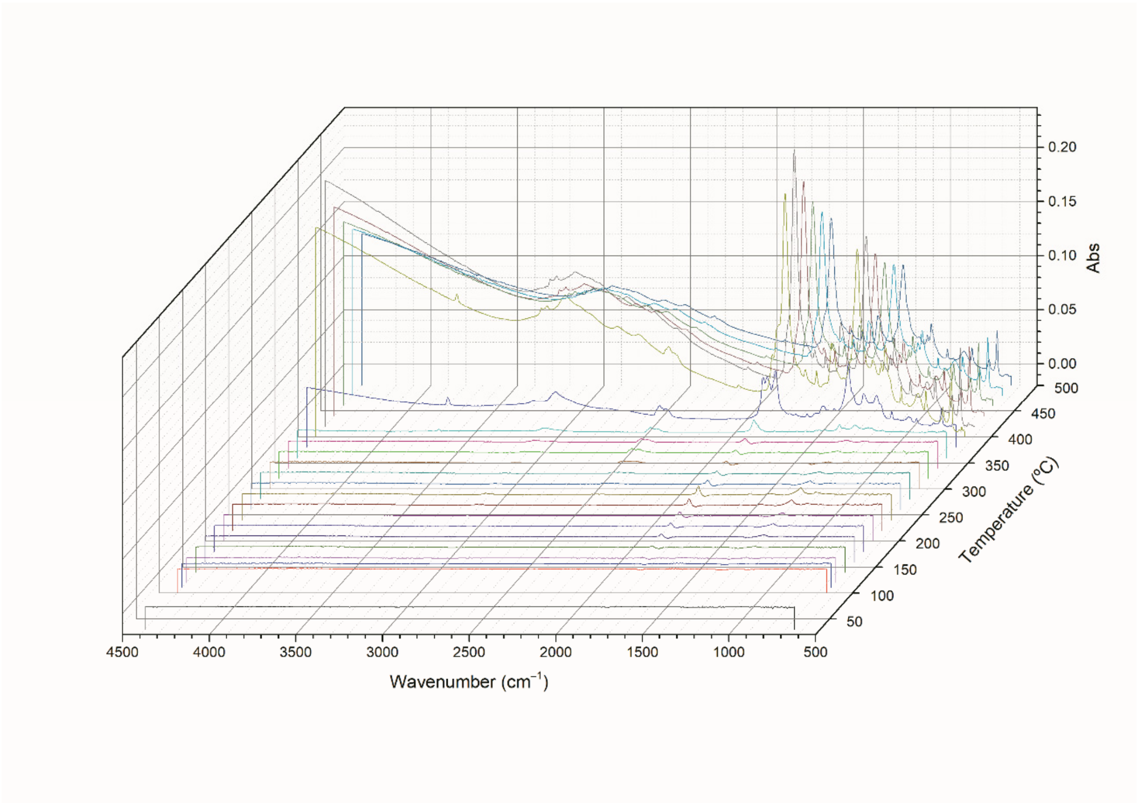

Supplement: Supplementary file 1 [file materials-15-01226-s001.zip › materials-1526891-supplementary.pdf]
